# Modeling Approach to Calculate the Orientation of Liquid Crystal Polymers in a Flow Channel Under Varying Boundary Conditions

**DOI:** 10.3390/polym17162209

**Published:** 2025-08-13

**Authors:** Gernot Zitzenbacher

**Affiliations:** School of Engineering, University of Applied Sciences Upper Austria, Stelzhamerstr. 23, 4600 Wels, Austria; g.zitzenbacher@fh-wels.at; Tel.: +43-(0)50804-44520

**Keywords:** liquid crystal polymers, thermoplastic, orientation, modeling, wall slip, slit die, polymer rheology

## Abstract

Thermotropic liquid crystal polymers comprise rigid chain segments called mesogens. This study presents a modeling approach to simulate the orientation of these mesogens in a flow channel with a rectangular cross section under no slip and wall slip boundary conditions. Rigid rods with finite length and an initial orientation are proposed. The interactions between the velocity field in the flow channel and these rods are modeled to simulate orientation. Moreover, a highly oriented boundary layer can be simulated. Orientation occurs in the flow direction close to the die wall under the no slip condition due to the high shear rate. As the distance from the die wall increases, the orientation decreases. Wall slip effectuates a more uniform orientation and causes a delay in the development of the highly oriented boundary layer. The thickness profile of this layer exhibits a shape that is analogous to that of a root function. To ensure products with high mechanical properties, it is essential to orient the mesogens at a high level in the die during manufacturing. The presented model enables the prediction of orientation in the flow channel. Therefore, this model is a useful tool to design the process in the right way to reach this goal.

## 1. Introduction

Liquid crystal polymers (LCPs) are materials that are in a thermodynamically stable intermediate phase, the so-called mesophase. This mesophase shows crystal-typical order structures in a flow, which are caused by so-called mesogens. Mesogens are linear molecule segments that are part of the polymer’s molecule structure [1,2].

With respect to the position of the mesogens, LCPs can be distinguished into different categories, namely main-chain LCPs, side-chain LCPs, main-chain/side-chain LCPs, and crosslinked LCPs. In main-chain LCPs, the mesogens are in the polymer main chain, and in side-chain LCPs, the mesogens are attached to the main chain as side groups. In addition, main-chain/side-chain LCPs with the mesogens acting as components of both main chains and side chains and crosslinked LCPs with the mesogens incorporated into a polymer network are known. In main-chain LCPs, the mesogens are incorporated between interference points in the main chain. In side-chain LCPs, they are grafted onto meltable macromolecules via spacers. LCPs with spacers or interfering points can be melted and processed and are referred to as thermotropic LCPs. If the main chain consists only of rigid segments, these LCPs cannot be melted and can only be processed in solution. These are referred to as lyotropic LCPs [3,4].

In addition to the molecular structure, the properties of thermotropic LCP parts are significantly influenced by morphology. In the mesophase, the mesogens are oriented during processing in the molten state and remain in this alignment in the solidified part. These rigid chain segments form a highly ordered structure, which is embedded in a “matrix” of the same material. This phenomenon is also known as self-reinforcement. As the improvement in properties only has an effect in the direction of orientation, the manufactured parts or products exhibit anisotropic properties. The type and strength of the orientation are determined by the shear and tensile stresses that occur during processing [5,6]. The oriented rigid chain segments act in a similar way to fibers in composite materials, which enhances the mechanical part properties [7,8,9].

To obtain high shear orientation in processing thermotropic LCPs, particular consideration has to be given to the design of dies and molds and to optimizing processing conditions. That should be carried out in such a manner that high shear rates for long shearing times are guaranteed [10]. For this purpose, the rheology of LCPs is very important and needed as an enabler for the right die and mold design.

The viscosity curve of thermotropic LCPs differs from that of conventional thermoplastic polymers. It is reported that the viscosity has three regions [11]. These polymers exhibit shear-thinning behavior in regions I and III, which are the low and high shear rate regions, respectively. Region II (the intermediate shear rate range) is the region of a Newtonian plateau [11,12,13]. The three-region viscosity curve was not consistently observed for LCPs. Other studies reported that thermotropic LCPs exhibit shear-thinning behavior across the entire range of shear rates, from low to high. In this case, the viscosity curve could be described using either a power law or a cross model [14,15,16].

Thermotropic LCPs can be processed with methods that are used for conventional thermoplastic polymers, such as injection molding, extrusion, melt spinning, or additive manufacturing.

The main focus of application is injection molding, where thin-walled parts with high stability and long flow paths for electrical devices and medical applications can be manufactured. Moreover, burr formation in the injection molding process is very low [17,18,19].

LCPs are also used in film extrusion, where the orientation of the mesogens depends on the type of process and die used. The manufactured LCP film is highly anisotropic when the flat film extrusion process is employed. Here, the mesogens are mainly oriented in the machine direction. This makes it easy for cracks to form in the film when a load is applied in the transverse direction. A less anisotropic LCP film with biaxially oriented mesogens can be manufactured using the blown film extrusion process with a counter-rotating core and mandrel in the blown film die [20,21]. Cast and blown LCP/PE films containing 10% LCP have a significantly higher elastic modulus than pure PE films [22,23]. Biaxially oriented blown LCP films are used in 5G applications, for example, in [24].

Fused filament fabrication (FFF) can be used to manufacture complex parts with a thin-walled structure and high mechanical properties. For example, it is intended to expand its scope of applicability to structural parts for space applications [25]. It is reported that the Young’s modulus and strength of the extruded filaments increase with a smaller nozzle diameter due to the increased fraction of oriented LCPs in the 3D printer [26]. Other authors observed that also a disorientation in the nozzle of the 3D printer can take place. They used drawn filaments with a high orientational order in the extrusion direction. After passing through the nozzle of the 3D printer, the initial high orientation was lost, and the melt was partly disoriented [27].

In this study, a new approach is presented to predict the flow orientation of mesogens in a flow channel with a rectangular cross section, considering no slip or wall slip conditions at the die wall. For this purpose, a combination of shear flow and slip flow is assumed. The shear thinning behavior of the polymer melt is taken into account using a power law model. In this model, the mesogens are simplified as rigid rods with finite length and an initial orientation in the flow field. These rigid rods undergo a differential translational movement in the flow direction during a differential time step. Additionally, due to the velocity distribution and local shear rate, a differential rotation takes place. Equations are obtained that enable the calculation of the orientation dependent on the position in the die. The proposed model is analyzed through example evaluations of the orientation of the rigid rods within the die. Furthermore, a method for calculating the thickness of a highly oriented boundary layer is presented.

## 2. Modeling

### 2.1. Basic Orientation Model

**Figure 1:**
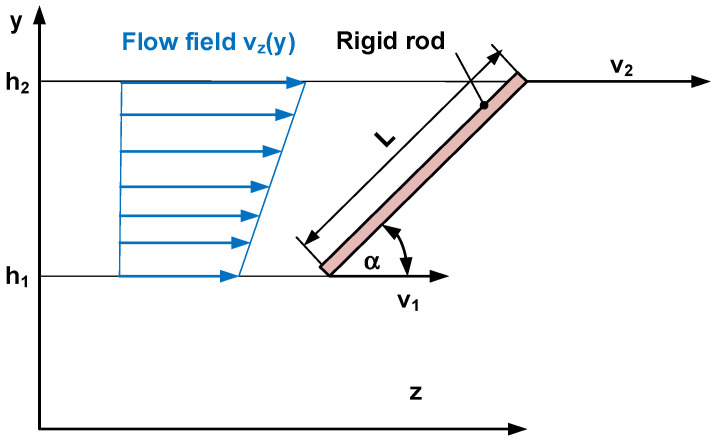
Modeling approach of the rigid rod in the flow velocity field with the orientation angle α, the positions *h*_1_ and *h*_2_, and the velocities *v*_1_ and *v*_2_ of the lower and the upper end, respectively.

As a modeling approach, a rigid rod is assumed in the flow field (see Figure 1). The interactions between the rigid rod and the flow velocity field are considered, and the orientation of the rod is calculated. It is assumed that the obtained orientation is similar to the mesogen orientation of a thermoplastic liquid crystal polymer. The further assumptions and prerequisites of the orientation model are as follows:

- The rigid rod has the length L and the orientation angle *α* in the flow. The dimensions of the rod are very small compared to the geometry of the flow channel. The positions of the top and the lower end of the rod are h_2_ and h_1_, respectively.
- Interactions between the rods are neglected.
- A laminar flow is assumed.
- A steady state and fully developed flow is assumed.
- Gravity and inertia forces are neglected.
- The flow takes place in a flow channel with a rectangular cross section with the die height *H*, which is much smaller than the die width *W*. In this case, the influence of the sidewalls can be neglected. Consequently, the problem can be simplified as a flow between two parallel plates with infinite lateral extension. The flow velocity field is one-dimensionally modeled in the *yz*-plane.

The position of the top end *h*_2_ is expressed relative to the position of the lower end *h*_1_ of the rod by

(1) $$h_{2}=h_{1}+Lsin\alpha .$$

The rigid rod is much smaller in size than the flow channel geometry. Therefore, a constant shear rate is assumed along the rigid rod, and the velocity *v*_2_ of the top end is

(2) $$v_{2}=v_{1}+\dot{\gamma }Lsin\alpha ,$$

where *v*_1_ is the velocity of the lower end and $\dot{\gamma }$ is the shear rate along the rod.

To calculate the rod’s orientation, the velocity difference ∆*v_z_* between the top and the lower ends in flow direction *z* is required:

(3) $$∆v_{z}=v_{2}-v_{1}=\dot{\gamma }Lsin\alpha .$$

The velocity of the center of the rod *v_m_* is used to calculate the axial movement of the rod in the flow. It is the average of the velocity at the top and at the lower end:

(4) $$v_{m}=\frac{v_{1}+v_{2}}{2}=v_{1}+\frac{\dot{\gamma }Lsin\alpha }{2}.$$

**Figure 2:**
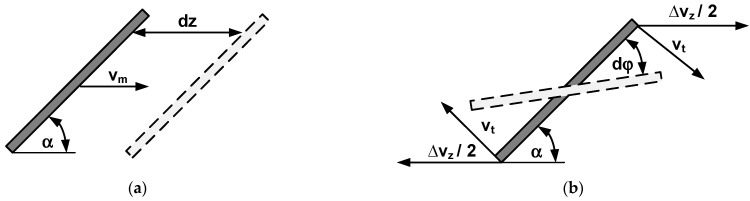
Interactions of the rigid rod with the flow velocity field within a differential time step *dt*: (**a**) differential movement in the flow direction; (**b**) differential rotation of the rod.

A differential time step *dt* is considered. During the time step, the rigid rod undergoes a differential movement *dz* in the flow direction (see Figure 2a):

(5) $$dz=v_{m}dt=\left(v_{1}+\frac{\dot{\gamma }Lsin\alpha }{2}\right)dt.$$

The flow field interacts with the rod. If the velocity were constant, there would only be axial movement with the flow. However, it should be noted that the flow velocity of the polymer melt is not constant in the slit die flow channel. The flow velocity depends on the *y*-coordinate direction. Therefore, the rod rotates due to the varying velocities within the flow field. Within a differential time step *dt*, the change of the rotation angle is *dφ* (see Figure 2b). The tangential velocity *v_t_* of the rod’s ends is

(6) $$v_{t}=\frac{∆v_{z}}{2}sin\alpha =\frac{\dot{\gamma }L{sin}^{2}\alpha }{2}.$$

A differential change in the rotation angle *φ* occurs during rotation within the time step *dt*. The differential arc length of the top and lower ends, calculated from the rotation angle *φ* and from tangential velocity *v_t_*, must be equal:

(7) $$-\frac{L}{2}d\varphi =v_{t}dt.$$

The differential change in orientation angle *α* is obtained from the differential change in rotation angle *φ*:

(8) $$d\alpha =\frac{d\varphi }{2}.$$

Using Equations (6) and (8) in Equation (7) gives

(9) $$-Ld\alpha =\frac{\dot{\gamma }L}{2}{sin}^{2}\alpha dt.$$

The separation of Equation (9) yields

(10) $$\int \frac{d\alpha }{{sin}^{2}\alpha }=-\frac{1}{2}\int \dot{\gamma }dt.$$

The shear rate remains constant along the rod’s flow path in a fully developed flow through a slit die. Therefore, the integration of Equation (10) adds up to

(11) $$cot\alpha =\frac{\dot{\gamma }t}{2}+cot\alpha_{0}.$$

Finally, the orientation angle *α* of the rigid rod is obtained as a function of time *t*:

(12) $$\alpha \left(t\right)=arccot\;\left(\frac{\dot{\gamma }t}{2}+cot\alpha_{0}\right).$$

*α*_0_ (rad) is the initial orientation angle, which is considered as the initial condition in Equation (12) for *t* = 0. An alignment in the flow direction corresponds to an orientation angle *α* of 0. An angle *α* of *π*/2 corresponds to an orientation that is perpendicular to the flow.

Furthermore, a degree of orientation *D* is defined as characterizing parameter

(13) $$D=1-\frac{2\alpha }{\pi }.$$

*D* is equal to 0 when the orientation angle is *π*/2, and *D* is equal to 1 when the alignment is completely in the flow direction.

### 2.2. Wall Adherence in the Flow Channel (No Slip Condition)

The model is derived for slit dies, where the die width *W* is much larger than the die height *H*. In this case, the influence of the side walls can be neglected. Furthermore, polymer melt adherence to the wall is assumed. The coordinate system is located in the center of the die. The *z*-direction is the flow direction, and the *y*-direction is normal to *z* (the shear direction). Then, the simplified equation of motion has to be solved:

(14) $$0=-\frac{\partial p}{\partial z}+\frac{\partial \tau_{yz}}{\partial y},$$

where *p* is the pressure and $\tau_{yz}$ is the shear stress component. The shear stress distribution in the slit die can be obtained by solving the differential Equation (14).

The shear thinning behavior of the polymer melt is described using a power law model

(15) $$\tau_{yz}=K\cdot {\left(\frac{\partial v_{z}}{\partial y}\right)}^{n},$$

where *K* is the consistency of the power law model and *n* is the power law index. Therefore, the shear rate $\dot{\gamma }$ in a slit die is obtained using Equation (15) and the solution of Equation (14):

(16) $$\dot{\gamma }\left(y\right)=\frac{\partial v_{z}}{\partial y}={\left(\frac{∆p}{l}\frac{1}{K}\right)}^{\frac{1}{n}}y^{\frac{1}{n}},$$

where ∆*p* is the pressure loss and *l* is the length of the die. Integrating Equation (16) and considering the no slip boundary condition leads to the flow velocity *v_z_* in the slit die:

(17) $$v_{z}\left(y\right)={\left(\frac{∆p}{l}\frac{1}{K}\right)}^{\frac{1}{n}}\cdot \frac{n}{n+1}\cdot \left[{\left(\frac{H}{2}\right)}^{\frac{1}{n}+1}-y^{\frac{1}{n}+1}\right].$$

Equations (16) and (17) and the residence time *t*

(18) $$t=\frac{z}{v_{z}\left(y\right)}$$

are inserted into Equation (12). This adds up to the orientation angle *α* in a flow through a slit die as a function of *y* and *z*, taking wall adherence into account:

(19) $$\alpha \left(y,z\right)=arccot\left\{\frac{1}{2}\cdot \frac{n+1}{n}\cdot \frac{z}{y}\cdot {\left[{\left(\frac{H}{2y}\right)}^{\frac{1}{n}+1}-1\right]}^{-1}+cot\alpha_{0}\right\}.$$

In addition, a highly oriented boundary layer is introduced, with a degree of orientation *D* of

(20) $$D\geq D_{b}.$$

The critical orientation value *D_b_* is chosen to be 0.99 for further evaluation.

Both the critical orientation *D_b_* and the initial orientation *D*_0_ are inserted in Equation (19) to obtain

(21) $$\left(1-D_{b}\right)\frac{\pi }{2}=arccot\left\{\frac{1}{2}\cdot \frac{n+1}{n}\cdot \frac{z_{b}}{y}\cdot {\left[{\left(\frac{H}{2y}\right)}^{\frac{1}{n}+1}-1\right]}^{-1}+cot\left[\left(1-D_{0}\right)\frac{\pi }{2}\right]\right\}.$$

Then, *z_b_* is expressed explicitly from Equation (21), enabling the calculation of the border of the highly oriented boundary layer:

(22) $$z_{b}=\frac{2n}{n+1}\cdot \left\{cot\left[\left(1-D_{b}\right)\frac{\pi }{2}\right]-cot\left[\left(1-D_{0}\right)\frac{\pi }{2}\right]\right\}\cdot y\left[{\left(\frac{H}{2y}\right)}^{\frac{1}{n}+1}-1\right].$$

### 2.3. Wall Slip in the Flow Channel

When wall slip is assumed in the flow through a slit die, Equation (16) is solved by considering a slip velocity *vs* a boundary condition at the die surface. Then the flow velocity *v_z_* is

(23) $$v_{z}\left(y\right)={\left(\frac{∆p}{l}\frac{1}{K}\right)}^{\frac{1}{n}}\cdot \frac{n}{n+1}\cdot \left[{\left(\frac{H}{2}\right)}^{\frac{1}{n}+1}-y^{\frac{1}{n}+1}\right]+v_{s}.$$

The shear rate $\dot{\gamma }\;$ in the flow with wall slip is obtained from Equation (23) through derivation:

(24) $$\dot{\gamma }\left(y\right)={\left(\frac{∆p}{l}\frac{1}{K}\right)}^{\frac{1}{n}}y^{\frac{1}{n}}.$$

Inserting Equations (18), (23) and (24) in Equation (12) accounts for

(25) $$\alpha \left(y,z\right)=arccot\left\{\frac{1}{2}\cdot \frac{{zy}^{\frac{1}{n}}}{\frac{n}{n+1}\cdot \left[{\left(\frac{H}{2}\right)}^{\frac{1}{n}+1}-y^{\frac{1}{n}+1}\right]+v_{s}{\cdot \left(\frac{∆p}{l}\frac{1}{K}\right)}^{-\frac{1}{n}}}+cot\alpha_{0}\right\}\;.$$

The volume flow rate *Q* is obtained by integrating the flow velocity *v_z_* (Equation (23)) over the cross-sectional area of the die. It can be expressed as the sum of the volume flow rates due to shear flow *Q_v_* and slip flow *Q_s_*:

(26) $$Q=Q_{v}+Q_{s}.$$

The volume flow rate due to shear flow *Q_v_* in a slit die is

(27) $$Q_{v}=2W{\cdot \left(\frac{∆p}{l}\frac{1}{K}\right)}^{\frac{1}{n}}\cdot \frac{n}{2n+1}\cdot {\left(\frac{H}{2}\right)}^{\frac{1}{n}+2}.$$

The volume flow rate due to wall slip *Q_s_* is

(28) $$Q_{s}=WHv_{s}.$$

From Equation (27) the following expression is obtained:

(29) $${\left(\frac{∆p}{l}\frac{1}{K}\right)}^{-\frac{1}{n}}=\frac{2W}{Q_{v}}\cdot \frac{n}{2n+1}\cdot {\left(\frac{H}{2}\right)}^{\frac{1}{n}+2}.$$

Equation (29) and the expression for wall slip velocity obtained from Equation (28) are inserted in Equation (25), which accounts for

(30) $$\alpha \left(y,z\right)=arccot\left\{\frac{1}{2}\cdot \frac{{zy}^{\frac{1}{n}}}{\frac{n}{n+1}\cdot \left[{\left(\frac{H}{2}\right)}^{\frac{1}{n}+1}-y^{\frac{1}{n}+1}\right]+\frac{Q_{v}}{Q_{s}}{\cdot \frac{n}{2n+1}\cdot \left(\frac{H}{2}\right)}^{\frac{1}{n}+1}}+cot\alpha_{0}\right\}\;.$$

The fraction of slip flow *f_s_* is introduced:

(31) $$f_{s}=\frac{Q_{s}}{Q}.$$

Inserting the fraction of slip flow *f_s_* (Equation (29)) and the volume flow rate due to shear flow *Q_V_* (Equation (26)) into Equation (30) yields the orientation angle *α* for a superposition of slip and shear flow:

(32) $$\alpha \left(y,z\right)=arccot\left\{\frac{1}{2}\cdot \frac{z}{y}\cdot {\left\{\frac{n}{n+1}\cdot \left[{\left(\frac{H}{2y}\right)}^{\frac{1}{n}+1}-1\right]+\frac{f_{s}}{{1-f}_{s}}{\cdot \frac{n}{2n+1}\cdot \left(\frac{H}{2y}\right)}^{\frac{1}{n}+1}\right\}}^{-1}+cot\alpha_{0}\right\}\;.$$

The critical orientation *D_b_* and the initial orientation *D*_0_ are now inserted into Equation (32) to obtain

(33) $$\left(1-D_{b}\right)\frac{\pi }{2}=arccot\left\{\frac{1}{2}\cdot \frac{z_{b}}{y}\cdot {\left\{\frac{n}{n+1}\cdot \left[{\left(\frac{H}{2y}\right)}^{\frac{1}{n}+1}-1\right]+\frac{f_{s}}{{1-f}_{s}}{\cdot \frac{n}{2n+1}\cdot \left(\frac{H}{2y}\right)}^{\frac{1}{n}+1}\right\}}^{-1}+cot\left[\left(1-D_{0}\right)\frac{\pi }{2}\right]\right\}\;.$$

Then, *z_b_* is explicitly expressed from Equation (33), which enables the calculation of the border of the highly oriented boundary layer under the consideration of wall slip:

(34) $$z_{b}=2n\cdot \left\{cot\left[\left(1-D_{b}\right)\frac{\pi }{2}\right]-cot\left[\left(1-D_{0}\right)\frac{\pi }{2}\right]\right\}\cdot y\left\{\frac{1}{n+1}\cdot \left[{\left(\frac{H}{2y}\right)}^{\frac{1}{n}+1}-1\right]+\frac{f_{s}}{{1-f}_{s}}{\cdot \frac{1}{2n+1}\cdot \left(\frac{H}{2y}\right)}^{\frac{1}{n}+1}\right\}.$$

## 3. Results

### 3.1. Orientation Angle and Degree of Orientation

**Figure 3:**
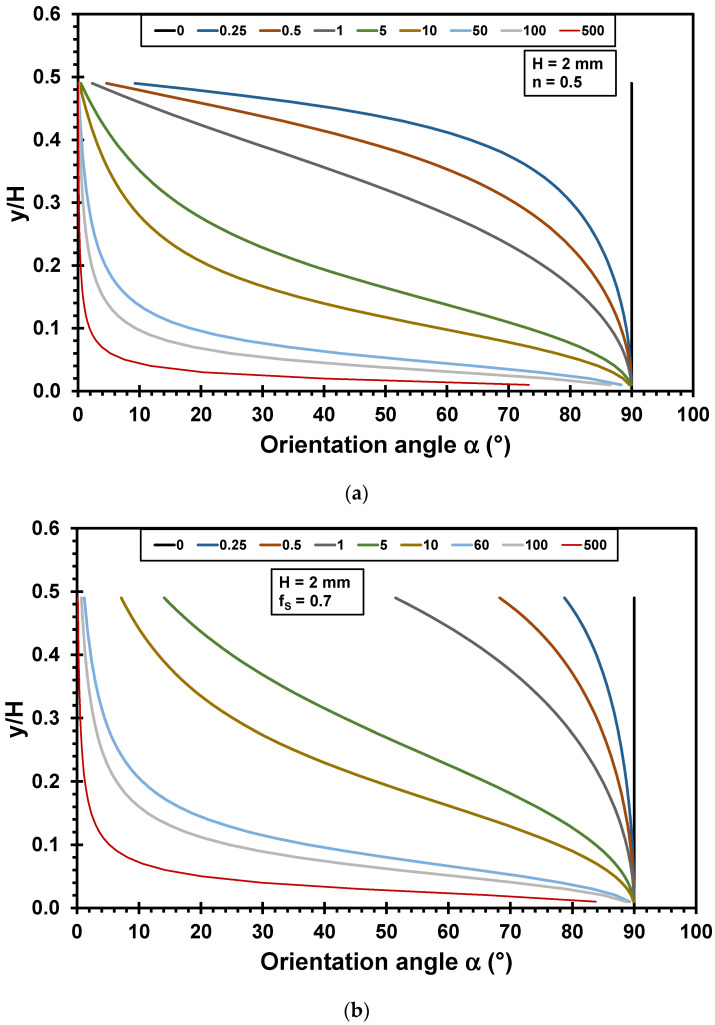
Orientation angle α in a slit die as a function of *y*/*H* for different positions *z* (in mm) (die height *H* = 2 mm, power law exponent *n* = 0.5): (**a**) no slip; (**b**) wall slip (fraction of wall slip *f_s_* 0.7).

Figure 3 shows an example evaluation of Equation (19) (no slip) and Equation (32) (wall slip) for the calculation of the evolution of the orientation angle in the slit die. The calculations were performed using a power law exponent *n* of 0.5, a slit die height *H* of 2 mm, and an initial orientation angle *α*_0_ of 90°. The fraction of slip flow *f_s_* is set to 0.7, respectively.

The orientation angle α is plotted as a function of *y*/*H* for different axial positions *z*. In the case of no slip (see Figure 3a), the orientation angle *α* rapidly decreases close to the die wall. In the center of the die, the orientation occurs more slowly. After a long flow path (*z* = 500 mm), almost the entire material is aligned in the flow direction. Only in the center of the die is a lower-oriented region, and at the position *y*/*H* = 0, no orientation is observed. In the case of wall slip (see Figure 3b), the decrease in the orientation angle close to the die wall is slower. After a long flow path (*z* = 500 mm), the distribution of the orientation angle *α* is similar to that using the no slip condition.

**Figure 4:**
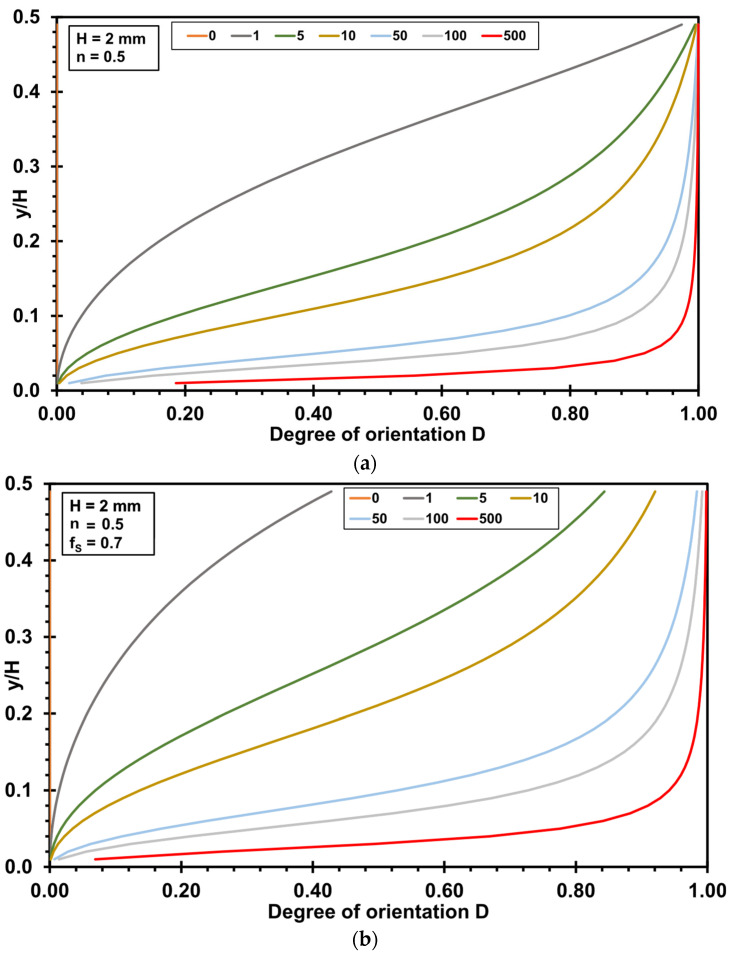
Degree of orientation *D* in a slit die as a function of *y*/*H* for different positions *z* (in mm) (die height *H* = 2 mm, power law index *n* = 0.5): (**a**) no slip; (**b**) wall slip (fraction of wall slip *f_s_* 0.7).

When evaluating the degree of orientation *D* in this example evaluation instead of the orientation angle *α*, an orientation behavior similar to that in Figure 3 is obtained. However, clearer evidence is visible when using the degree of orientation, as no orientation is accounted for at a value of 0, and a complete alignment in the flow direction is represented by a value of 1 (see Figure 4).

### 3.2. Influence of Rheological Parameters on the Degree of Orientation

**Figure 5:**
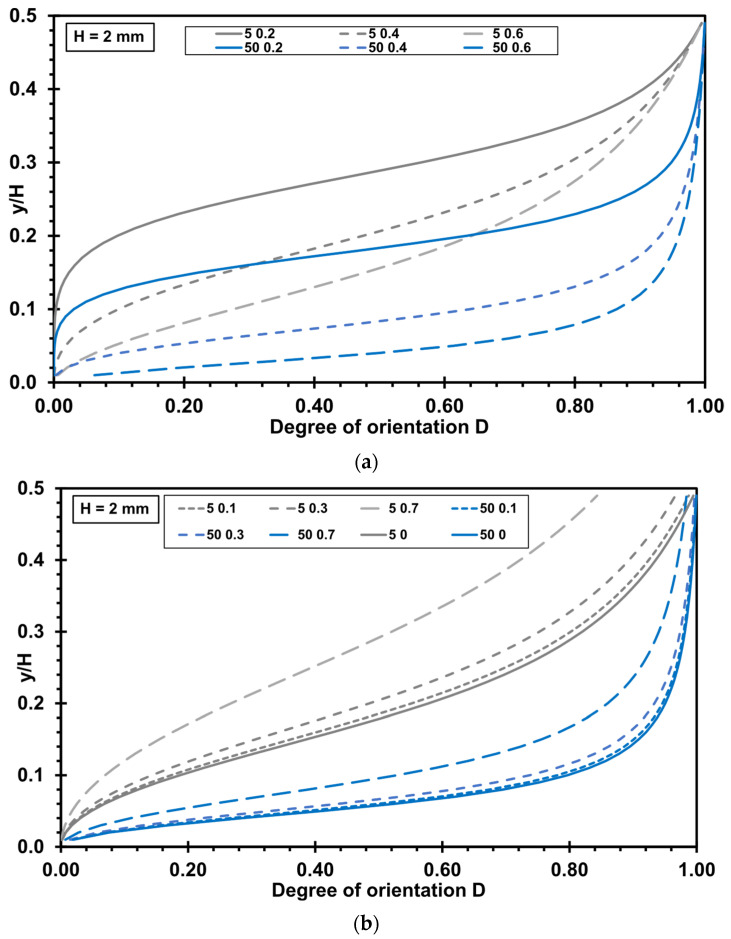
Influence of rheological parameters on the degree of orientation *D* in a slit die as a function of *y*/*H* for different axial positions *z* = 5 mm (grey lines) and *z* = 50 mm (blue lines) (die height *H* = 2 mm): (**a**) influence of the power law exponent *n* under no slip condition (*n* = 0.2, 0.4, and 0.6); (**b**) influence of the fraction of wall slip *f_s_* (*f_s_* = 0, 0.1, 0.3, 0.7; power law exponent *n* = 0.5).

The flow velocity profile in a slit die is influenced by the power law exponent *n*, as well as by a slip or no slip boundary condition [28]. Consequently, the influence of these parameters on orientation in a slit die is significant. Figure 5 shows the effect of the power law exponent *n* under no slip condition (Figure 5a) and the effect of the fraction of wall slip *f_s_* under slip condition (Figure 5b) on the degree of orientation *D* in a flow through a slit die.

Assuming a low power law exponent *n* of 0.2 leads to an unoriented flow region (between *y*/*H* = 0 and approximately *y*/*H* = 0.2) at the beginning of the flow (*z* = 5 mm). Towards the die wall, orientation takes place, but even after a certain flow path, there remains a larger unoriented flow region in the center of the die. With increasing power law exponent *n* (0.4 and 0.6), the orientation is higher and takes place faster in the center of the die. Furthermore, the orientation is more uniform along the die height (*z* = 50 mm).

Wall slip causes a delay in the orientation within the flow. This means that a longer flow path is required compared to the no slip condition to achieve a similar degree of orientation. Furthermore, the higher the fraction of wall slip *f_s_*, the lower is the orientation at the die wall at the beginning of the flow (*z* = 5 mm). After a longer flow path (*z* = 50 mm), however, the differences in orientation for different fractions of wall slip *f_s_* decrease, with the orientation curves shifting closer together.

### 3.3. Highly Oriented Boundary Layer

**Figure 6:**
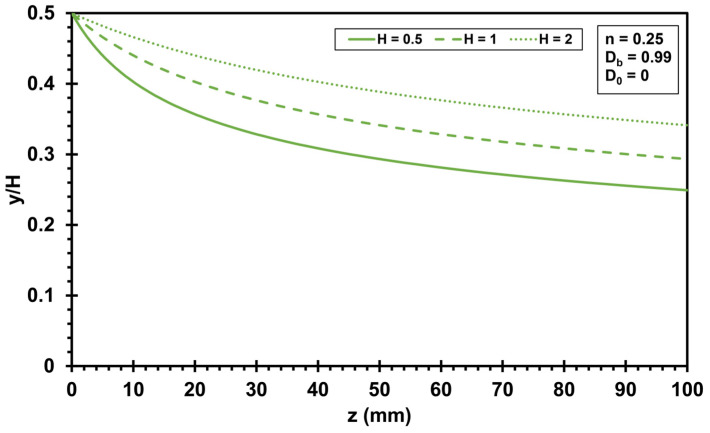
Influence of slit die height *H* on the evolution of the highly oriented boundary layer under no slip condition (*H* = 0.5 mm, 1 mm, and 2 mm; power law exponent *n* = 0.25, initial orientation *D*_0_ = 0, critical orientation value *D_b_* = 0.99).

The orientation is very fast close to the die, but a much longer flow path is needed for alignment in the flow direction towards the center of the die. Therefore, a highly oriented boundary layer develops in the flow close to the die wall. The evolution of this highly oriented boundary layer is visualized by evaluating Equation (22) (no slip condition) and Equation (34) (slip boundary condition). Figure 6 shows how the die height influences the evolution of the highly oriented boundary layer, assuming a power law exponent *n* of 0.25. Generally, the thickness of the highly oriented boundary layer is zero at the beginning of the flow. As the flow path increases, the thickness of the boundary layer increases. The thickness of the highly oriented boundary layer increases in a shape similar to a root function as z increases. A die height *H* of 2 mm results in the thinnest highly oriented boundary layer at the end of the flow path (*z* = 100 mm). The smaller the die height, the smaller is the thickness of the highly oriented boundary layer (compare *H* = 0.5 mm and *H* = 1 mm).

**Figure 7:**
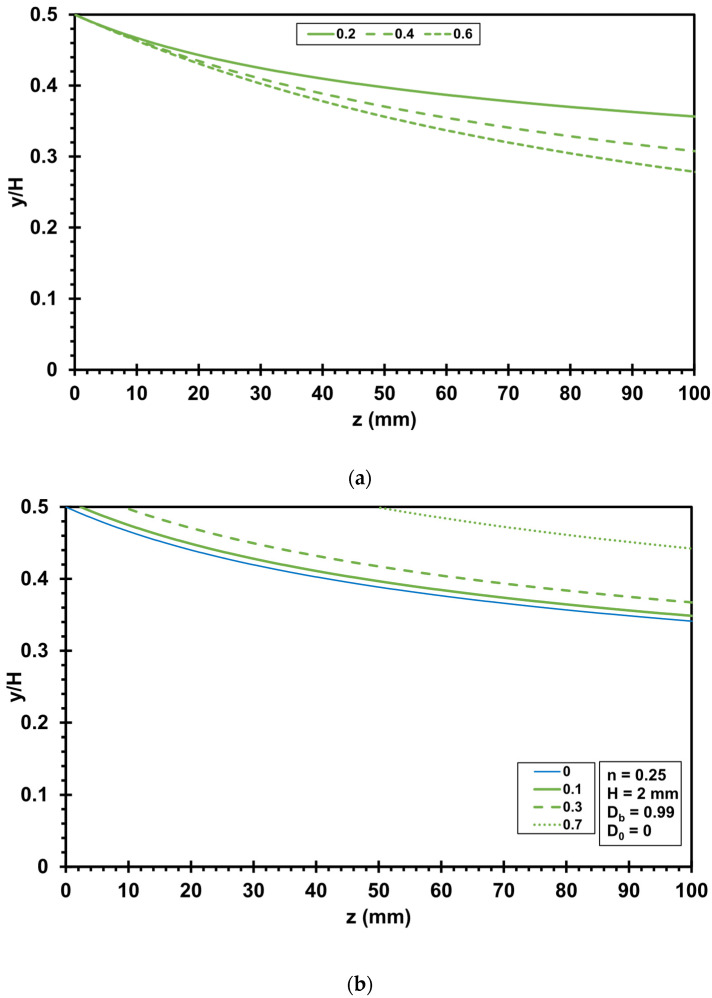
Influence of rheological parameters on the formation and evolution of the highly oriented boundary layer (initial orientation *D_0_* = 0, critical orientation value *D_b_* = 0.99, slit die height *H* = 2 mm): (**a**) influence of the power law index *n* (*n* = 0.2, 0.4, and 0.6); (**b**) influence of slip or no slip condition (*f_s_* = 0, 0.1, 0.3, 0.7).

Figure 7a illustrates the impact of the power law index on the evolution of the highly oriented boundary layer. The values of the power law index *n* are 0.2, 0.4, and 0.6, respectively. A higher power law index results in a faster increase in the thickness of the highly oriented boundary layer. Figure 7b shows the influence of wall slip, where the fraction of wall slip *f_s_* is 0, 0.1, 0.3, and 0.7, respectively. More wall slip results in less shear in the flow. As the fraction of wall slip *f_s_* increases (as shear decreases), the starting point in the flow channel of the highly oriented boundary layer shifts to higher values of *z*. Furthermore, wall slip reduces the thickness of the highly oriented boundary layer at the end of the flow channel.

## 4. Discussion

The proposed model (Equation (12)) predicts an increase in the degree of orientation D of the rigid rods in the flow direction, which is equivalent to a decrease in the orientation angle *α* as the product of shear rate $\dot{\gamma }$ and residence time *t* increases. This result is consistent with the known orientation behavior of thermotropic LCPs, as it has been reported that an increase in shear rate and shearing time enhances mesogen orientation [6,10].

When considering the flow through a slit die under the no slip condition, the velocity profile has a maximum value in the center of the die. Therefore, the shear rate is zero in the center of the die. As we move towards the die wall, the shear rate increases according to a root function that also depends on the power law exponent *n* (see Equation (16)). This explains the orientation, which is predicted by the model (Equation (19)). At the beginning of the flow, the orientation is zero in the center of the die (zero shear rate) and increases towards the die wall (region of higher shear rate). After a long flow path, which equates to a long residence time, the orientation is almost entirely in the flow direction. There remains no orientation in the flow direction (*α* = *π*/2) in the center of the die because the shear rate is zero there.

The existence of a highly oriented boundary layer is attributed to the high shear rate at the die wall. As residence time increases in the die, more and more polymer is oriented to a degree of orientation *D* ≥ *D_b_*. This results in an increase in thickness in the shape of a root function. The increase in thickness takes place faster for a lower die height *H*. This observation is explained by the increased shear rate in thin-walled flow channels, which also explains that LCPs are mainly suitable for thin-walled parts [17,18,19].

A lower power law exponent *n* increases the region of no orientation in the center of the die. This result is consistent with the influence of the power law exponent on the velocity profile and shear rate distribution. It has been reported that a decrease in the power law exponent results in a flat velocity profile in the center and a faster decrease in velocity towards the die wall [28]. This means that the region of no shear in the center of the die increases as the power law exponent decreases. This also explains why the thickness of the highly oriented boundary layer increases more slowly for lower power law exponents *n*.

Wall slip results in a delay in the orientation compared to pure shear flow. This means that a longer flow path or longer residence time is required to achieve the same orientation as with a no slip boundary condition. This effect is observed to increase with a larger fraction of wall slip. This delay can also be seen in the predicted thickness of the highly oriented boundary layer. As the fraction of wall slip increases, there is a delay in the formation and a decrease in thickness.

A previously performed study analyzed mesogen orientation of films and ribbons manufactured from a thermotropic LCP, namely Vectra A950 from Celanese Corporation, Irving, Texas, USA. The films were produced using two different methods: a flat film extrusion line and a high-pressure capillary rheometer equipped with a slit die. The orientation in the extruded products was determined using greyscale analysis in combination with optical microscopy [29]. The slit die used in this study in combination with the high-pressure capillary rheometer has a flow channel with a rectangular cross section. This means the flow channel geometry used in these experiments is the same as that of the proposed model.

Depending on the orientation of the mesogens, the cross section of the extruded products exhibits areas of different brightness. Brighter areas indicate a lower degree of orientation, whereas darker areas are more highly oriented. It has been reported that orientation-dependent light scattering causes these brightness differences, making the grey scale value a qualitative indicator of mesogen orientation [18,30,31].

It was observed that the extruded LCP ribbons exhibit a low-oriented core region in the center, which is attributed to the flow in the middle of the flow channel. The orientation increases towards the top and bottom sides of the ribbon. Additionally, a highly oriented boundary layer was identified [29]. Although no absolute orientation values were measured, the shape of the orientation profile experimentally obtained is comparable to that predicted by the model.

Future research will extend this orientation model to other flow geometries and more complex flows, such as the flow in a flat film extrusion die. It is also planned to incorporate the effects of extensional flow and relaxation.

## 5. Conclusions

This study presents a model that can be used to predict the orientation of rigid rods in a flow channel with a rectangular cross section under consideration of no slip or wall slip conditions. These rods model mesogens in thermotropic LCPs in a simplified manner. The proposed model enables the calculation of the orientation dependent on the position in the die. A degree of orientation is introduced, where a value of zero is equivalent to an orientation perpendicular to the flow and a value of one indicates a complete alignment in the flow direction. The influence of rheological parameters such as the power law index or wall slip and geometrical parameters such as the die height on the orientation are determined. Additionally, the existence of a highly oriented boundary layer in the flow is proposed, and a method to calculate its thickness is presented. This thickness increases with rising residence time in the flow channel, and the shape of the thickness profile is similar to a root function. Furthermore, the highly oriented boundary layer is significantly influenced by wall slip. Wall slip causes a delay in the development of this boundary layer and in the increase in thickness. The orientation predicted by this model is in good agreement with the orientation determined experimentally and reported in the literature.

In practical applications, achieving a high mesogen orientation in the die is essential for producing products with high mechanical properties. The presented model enables the prediction of orientation in the flow channel. Therefore, this model is a useful tool for designing processes to reach this goal.

## Data Availability

The original contributions presented in this study are included in this article. Further inquiries can be directed to the corresponding author.

## Nomenclature

The following abbreviations and symbols are used in this manuscript:

*α*	Orientation angle of the rigid rod
*α*_0_	Initial orientation angle of the rigid rod
$\dot{\gamma }$	Shear rate
*φ*	Rotation angle
$\tau_{yz}$	Shear stress component in z-direction (yz-plane)
*D*	Degree of orientation
*D*_0_	Initial degree of orientation
*D_b_*	Critical orientation value at the beginning of the highly oriented boundary layer
*f_s_*	Fraction of slip flow
*H*	Die height
*h*_1_	Position of the lower end of the rod
*h*_2_	Position of the top end of the rod
*K*	Consistency (power law model)
*n*	Power law index
*l*	Length of the die
*L*	Length of the rigid rod
LCP	Liquid crystal polymers
PE	Polyethylene
*p*	Pressure
∆*p*	Pressure loss
*v*_1_	Velocity of the lower end of the rod
*v*_2_	Velocity of the top end of the rod
∆*v_z_*	Velocity difference between the top and the lower end of the rod
*v_m_*	Velocity of the center of the rod
*v_t_*	Tangential velocity of the ends of the rod
*v_z_*	Flow velocity in the slit die
*v_s_*	Slip velocity at the die surface
*Q*	Volume flow rate
*Q_v_*	Volume flow rate due to shear flow
*Q_s_*	Volume flow rate due to slip flow
*W*	Die width
*x*	Coordinate direction, indifferent direction
*y*	Coordinate direction, shear direction
*z*	Coordinate direction, direction of the flow
*z_b_*	Position of the border of the highly oriented boundary layer
